# A case of retroperitoneal liposarcoma extending through the inguinal canal to the thigh and lesser trochanter

**DOI:** 10.1002/iju5.12600

**Published:** 2023-06-16

**Authors:** Takahiro Maekawa, Yoshiyuki Yamamoto, Taigo Kato, Koji Hatano, Atsunari Kawashima, Shinichiro Fukuhara, Ryoichi Imamura, Norio Nonomura

**Affiliations:** ^1^ Department of Urology Osaka University Graduate School of Medicine Suita Osaka Japan

**Keywords:** inguinal canal, psoas major muscles, quality of life, retroperitoneal liposarcoma, well‐differentiated liposarcoma

## Abstract

**Introduction:**

Liposarcoma is the most common retroperitoneal soft tissue tumor. Liposarcomas are often asymptomatic and are discovered after they become huge. Surgical resection is the first‐line treatment for retroperitoneal liposarcoma, but the surrounding organs are often resected with the liposarcoma.

**Case presentation:**

A man saw a hospital with a complaint of left lower abdominal distention, and a left retroperitoneal mass was noted on imaging examination. The patient was referred to our hospital. The mass extended from the retroperitoneum through the inguinal canal to the thigh and involved the femoral nerve and psoas major muscle. A well‐differentiated liposarcoma was suspected, and an open surgical resection was performed. Complete resection of a retroperitoneal liposarcoma extending to the thigh was achieved without postoperative complications.

**Conclusion:**

Treatment strategies for huge retroperitoneal liposarcomas are important to balance antitumor efficacy and postoperative quality of life.

**Keynote message**

Retroperitoneal liposarcomas that extend through the inguinal canal to the thigh and lesser trochanter are rare. In many cases, concurrent resection of the surrounding organs is performed. In this case, we could completely remove the tumor maintaining postoperative quality of life through appropriate treatment strategies of surgical methods.

## Introduction

Liposarcoma is a relatively frequent malignant soft tissue tumor with a predilection for the retroperitoneum and lower extremities.^1^, ^2^ There are generally no disease‐specific symptoms, and retroperitoneal liposarcomas are often detected after they become large. Enlarged liposarcomas rarely extend into the inguinal canal. Positive margins are a poor prognostic factor, and it is important to ensure adequate margins and consider the resection of other organs if necessary.^2^, ^3^, ^4^ Herein, we report a case of retroperitoneal liposarcoma extending through the inguinal canal to the thigh and lesser trochanter.

## Case presentation

**Fig. 1:**
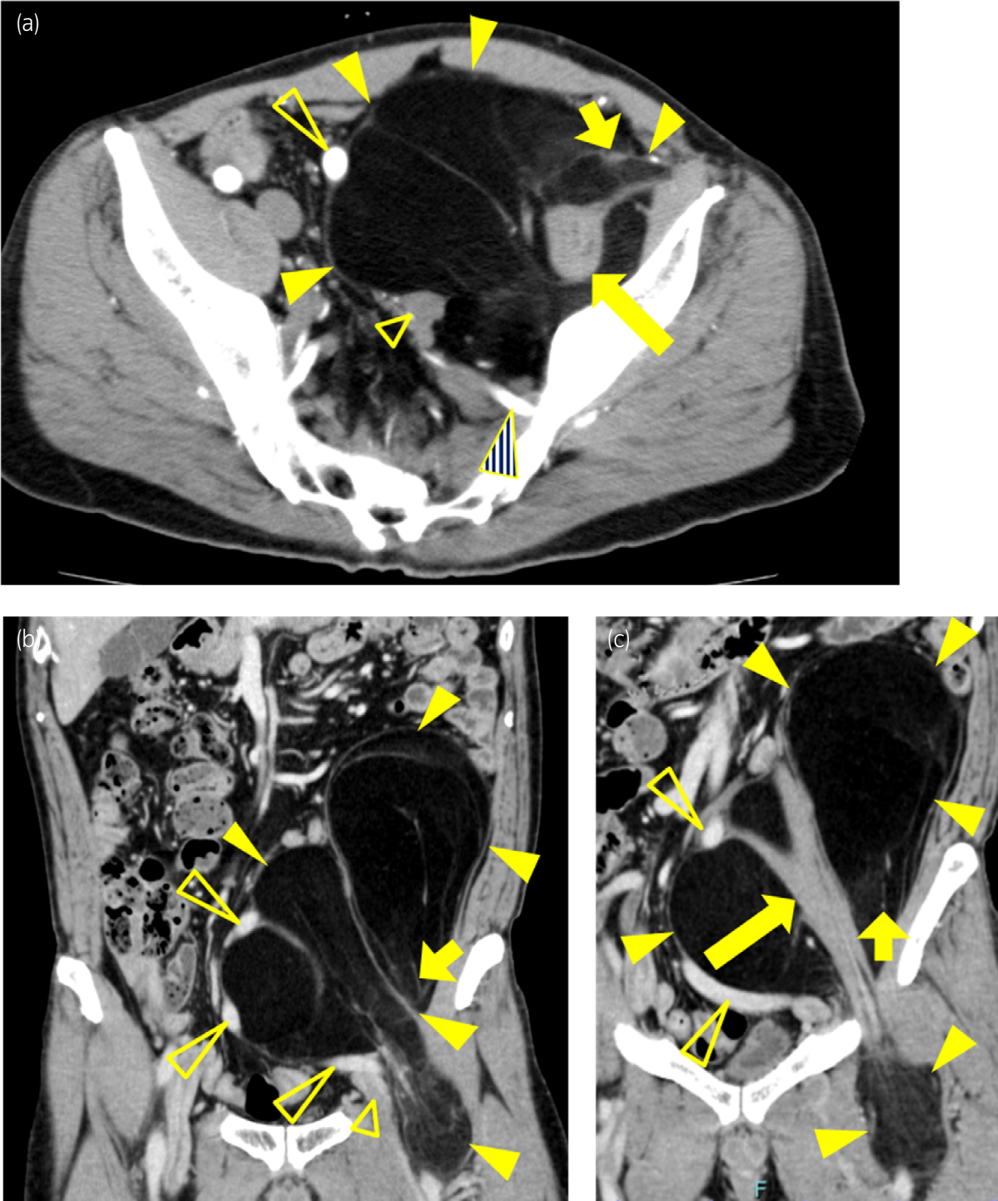
Enhanced CT scan showing a mass occupying the left retroperitoneal and left pelvic cavities extending through the inguinal canal to the thigh. (a) Horizontal section (b, c) Coronal section. Arrowheads, mass; long arrow, psoas major muscle; short arrow, femoral nerve; long open triangle, left external iliac artery; short open triangle, left external iliac vein; triangle with oblique lines, left internal iliac artery.

The patient was a 67‐year‐old man. He was referred to our department with a chief complaint of lower abdominal distention. On arrival at the hospital, Eastern Cooperative Oncology Group performance status was 0, and physical examination revealed only a palpable elastic hard mass in the left lower abdomen. Vital signs, neurological findings, and blood tests were normal. Contrast‐enhanced CT abdominal imaging showed a 16 × 11 × 28 cm long mass occupying the left retroperitoneal and pelvic cavities, extending through the left inguinal canal to the left thigh (Fig. 1a–c). CT imaging revealed that the interior of the mass was low‐density with a possible fatty component, and no metastasis to other organs or lymph nodes was observed. Based on the imaging findings, a well‐differentiated liposarcoma was suspected, and surgical resection was planned.

**Fig. 2:**
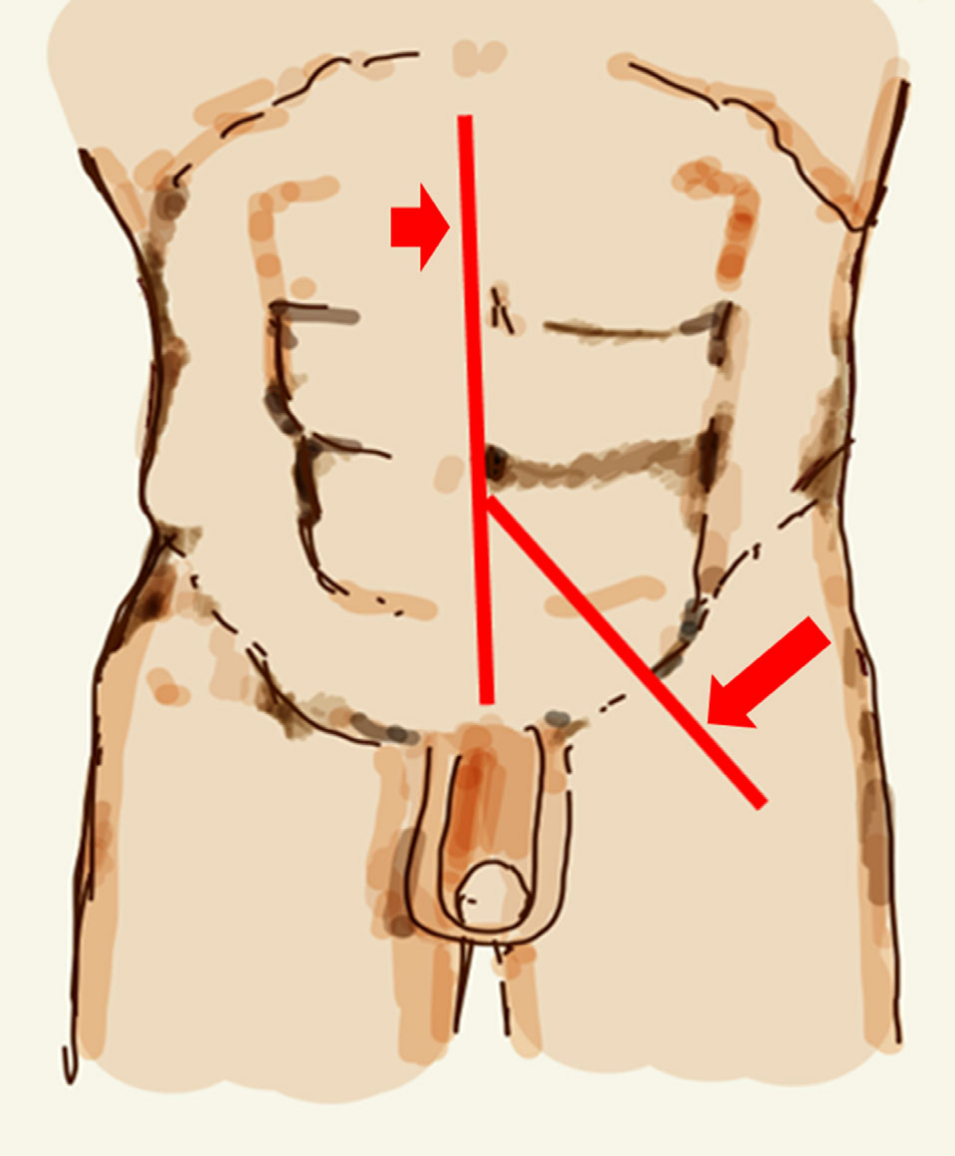
Diagram of median abdominal incision and incision to the left thigh. Short arrow, median abdominal incision; long arrow, incision to the left thigh.

**Fig. 3:**
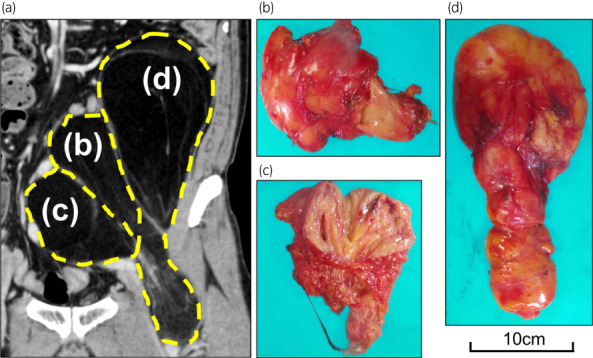
(a) Diagram of the position of the three pieces of tumor matched with coronal image of CT scan that was the same as Figure 1b. (b) to (d) in Figure 3a corresponded to those of the gross findings. (b–d) Macroscopic findings of the split surface of the excised specimen. The tumor was divided into three pieces. All figures were head‐side up and right‐side left. The 10 cm standard was applied to (b–d).

**Fig. 4:**
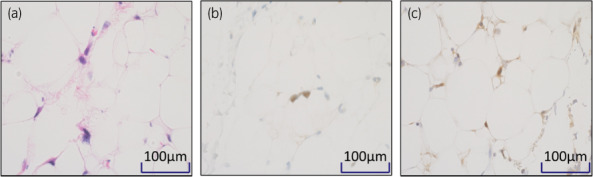
(a) Hematoxylin and eosin staining showing mature adipocytes with fibrovascular stroma and scattered atypical cells with pleomorphic swollen nuclei. (b, c) Immunohistochemical staining was positive for MDM2 (b) and Cdk4 (c).

The operation was started through a midline abdominal incision (Fig. 2), and adhesions between the tumor and surrounding tissue were mild. The left psoas major muscle, left external iliac artery, and left femoral nerve were visible through the tumor and could be preserved. The rectus abdominis muscle and inguinal ligament were incised by extending the skin incision to the thigh for ensuring good surgical vision (Fig. 2). The tumor was up to the level of the lesser trochanter, but all tumors were resectable. Since it was difficult to remove the tumor in one lump, it was divided into three pieces to preserve the psoas major muscle and femoral nerves (Fig. 3). Surgery time was 6 h and 26 min, and blood loss was 2140 mL. The main causes of bleeding were slow hemorrhage from the tumor and ascites/lymphatic fluid. The split surface of the excised specimen was yellowish‐white with some grayish‐white areas of fullness (Fig. 3b–d). The total tumor weight was 1660 g, and the maximal size of the resected tumor was 28 cm. On histopathology, hematoxylin and eosin staining showed mature adipocytes with fibrovascular stroma and scattered atypical cells with pleomorphic swollen nuclei (Fig. 4a). Immunohistochemical staining was positive for MDM2 (Fig. 4b) and Cdk4 (Fig. 4c); a well‐differentiated liposarcoma was diagnosed. Pathological grade using the FNCLCC (Fédération Nationale des Centers de Lutte Contre le Cancer) grading system^5^ was grade 1, and no obvious positive pathologic margins were observed. There were no postoperative motor or sensory deficits in the lower extremities. Currently, 3 years after surgery, no recurrence has been observed.

## Discussion

Liposarcoma is a nonepithelial malignant tumor of soft tissue origin and is the most common (about 40%–50%) retroperitoneal soft tissue tumor.^2^, ^6^ Liposarcomas occur most frequently in the extremities (50.9%), retroperitoneal cavity (23.2%), and trunk (9.6%).^1^ Retroperitoneal liposarcomas are most common in the 50s and 60s and are slightly more common in men.^3^, ^7^ Retroperitoneal liposarcomas rarely extend into the inguinal canal and present as an inguinal mass, as in this case.

Liposarcomas grow slowly and painlessly and are usually distensible. Since there are no specific symptoms, it is difficult to diagnose the disease in its early stage; it is often detected after the tumor has grown to a large size.^8^ Retroperitoneal liposarcomas are generally large at presentation, with nearly 50% being larger than 20 cm at diagnosis.^9^ The diagnosis is based on imaging findings, such as ultrasonography, CT, and magnetic resonance imaging. According to the new WHO classification in 2020, retroperitoneal liposarcomas are classified into five types based on pathological diagnosis: well‐differentiated liposarcoma, dedifferentiated liposarcoma, myxoid liposarcoma, pleomorphic liposarcoma, and myxoid pleomorphic liposarcoma.^10^ Prognosis varies greatly, depending on histology. The 5‐year survival rate is 60%–83% for all liposarcomas, and the prognosis is relatively good for the well‐differentiated and myxoid types, at 92%–93% and 92%, whereas the prognosis is poor for the pleomorphic and dedifferentiated types, at 20%–44% and 59%, respectively.^3^, ^11^ Regarding the association between surgical margin and prognosis, 3‐year survival rates are reported to be about 87%–90% in the surgical margins‐negative group, 70%–84% in the microscopically‐positive group, and 43%–51% in the grossly‐positive group.^3^, ^11^ Thus, it is important to aim for complete resection as much as possible in surgery for liposarcoma, including resection of surrounding organs.^2^, ^12^, ^13^

Giant retroperitoneal liposarcomas may be discovered in an inguinal hernia.^14^, ^15^ The diameter of retroperitoneal liposarcoma with inguinal extension averaged 27.9 cm, and the overall size was huge.^16^ Many cases required the concurrent resection of other organs such as kidneys, colon, and testes, and 67% of cases had recurrence within 1 to 20 months.^16^ Past reports of retroperitoneal liposarcoma with inguinal extension have mostly been in males.^14^, ^15^, ^16^ Inguinal hernias are more common in men^17^ because the inguinal canal is more fragile in men than in women. Therefore, it can be inferred that the inguinal extension of retroperitoneal liposarcomas is more common in males.

The tumor had extended to the thigh and lesser trochanter, so an additional incision to the thigh allowed complete resection of the tumor with good visual field. There were no preoperative findings suspicious of a dedifferentiated component in this case, and the adhesions to the psoas major muscle, femoral nerve, external iliac artery, and spermatic cord were mild, so the surgery was completed with these organs preserved. Moreover, since no component of a dedifferentiated tumor was estimated preoperatively, the tumor was removed in 3 parts to preserve the psoas major muscle and femoral nerve. An orchiectomy was planned for adhesion between the tumor and the spermatic cord, but since there was no adhesion, the spermatic cord and testis were spared. Liposarcoma has a high recurrence rate and requires regular monitoring.

In conclusion, we report a case of retroperitoneal liposarcoma extending through the inguinal canal to the thigh and lesser trochanter. The femoral nerve and psoas major muscle were spared, and the tumor was removed without compromising the postoperative quality of life. It is important to consider treatment strategies for huge liposarcomas, including preoperative preparation, to balance antitumor efficacy and postoperative quality of life.

## Author contributions

Takahiro Maekawa: Writing – original draft. Yoshiyuki Yamamoto: Writing – review and editing. Taigo Kato: Writing – review and editing. Koji Hatano: Writing – review and editing. Atsunari Kawashima: Writing – review and editing. Shinichiro Fukuhara: Writing – review and editing. Ryoichi Imamura: Writing – review and editing. Norio Nonomura: Writing – review and editing.

## Conflict of interest

The authors declare no conflict of interest.

## Approval of the research protocol by an Institutional Review Board

Not Applicable.

## Informed consent

Written informed consent was obtained from the patient for the publication of this case report and the accompanying images.

## Registry and the Registration No. of the study/trial

Not Applicable.

## Data Availability

Not Applicable.
